# Ultrasonography survey and thyroid cancer in the Fukushima Prefecture

**DOI:** 10.1007/s00411-013-0508-3

**Published:** 2014-01-09

**Authors:** Peter Jacob, Jan Christian Kaiser, Alexander Ulanovsky

**Affiliations:** Department of Radiation Sciences, Institute of Radiation Protection, Helmholtz Zentrum München-German Research Center for Environmental Health, Ingolstädter Landstraße 1, 85764 Neuherberg, Germany

**Keywords:** Fukushima, Radiation, Thyroid cancer, Ultrasonography

## Abstract

Thyroid cancer is one of the major health concerns after the accident in the Fukushima Dai-ichi nuclear power station (NPS). Currently, ultrasonography surveys are being performed for persons residing in the Fukushima Prefecture at the time of the accident with an age of up to 18 years. Here, the expected thyroid cancer prevalence in the Fukushima Prefecture is assessed based on an ultrasonography survey of Ukrainians, who were exposed at an age of up to 18 years to ^131^I released during the Chernobyl NPS accident, and on differences in equipment and study protocol in the two surveys. Radiation risk of thyroid cancer incidence among survivors of the atomic bombings of Hiroshima and Nagasaki and preliminary estimates of thyroid dose due to the Fukushima accident were used for the prediction of baseline and radiation-related thyroid cancer risks. We estimate a prevalence of thyroid cancer of 0.027 % (95 % CI 0.010 %; 0.050 %) for the first screening campaign in the Fukushima Prefecture. Compared with the incidence rate in Japan in 2007, the ultrasonography survey is predicted to increase baseline thyroid cancer incidence by a factor of 7.4 (95 % CI 0.95; 17.3). Under the condition of continued screening, thyroid cancer during the first fifty years after the accident is predicted to be detected for about 2 % of the screened population. The prediction of radiation-related thyroid cancer in the most exposed fraction (a few ten thousand persons) of the screened population of the Fukushima Prefecture has a large uncertainty with the best estimates of the average risk of 0.1–0.3 %, depending on average dose.

**Electronic supplementary material:**

The online version of this article (doi:10.1007/s00411-013-0508-3) contains supplementary material, which is available to authorized users.

## Introduction

Large amounts of radionuclides were released from the Fukushima Dai-ichi nuclear power station (NPS) in the aftermath of the 2011 Great East Japan earthquake, tsunami and reactor accident. Thanks to prompt actions of Japanese authorities, radiation exposures of the population were generally low. However, average thyroid doses of young children in some settlements were reported to be of the order of or even exceeding 100 mSv (WHO 2013). These dose estimates are based on measurements on environmental and food samples (WHO 2012). In contrast, measurements of incorporated ^131^I indicate that thyroid doses in the population were lower than the WHO estimate (Kamada et al. 2012; Tokonami et al. 2012; Kim et al. 2013; Matsuda et al. 2013).

There is concern about thyroid cancer induced by the accident at the Fukushima Dai-ichi NPS. The concern is caused by the massive increase in thyroid cancer among those who were highly exposed during childhood due to the Chernobyl accident (Kazakov et al. 1992; UNSCEAR 2011). In order to monitor thyroid cancer, periodic thyroid ultrasonography surveys have been introduced for all inhabitants of the Fukushima Prefecture who were 18 years old or younger at the time of the accident (Fukushima Medical University 2013a).

Thyroid cancer is a rare disease, although thyroid cancer incidence has increased worldwide during the previous decades. This increase might be related to improved diagnostics. No evidence for an influence of stable iodine supply could be demonstrated in a Danish study comparing thyroid cancer incidence before and after iodine supplementation (Bloomberg et al. 2012). Ultrasonography has a large potential of detecting so-called occult carcinoma that do not become clinically relevant during lifetime (Welch and Black 2010; Moynihan et al. 2012). The existence of such carcinoma has been demonstrated by autopsies with prevalence values ranging from 1.5 % in Greece (Delides et al. 1987) to 36 % in Finland (Harach et al. 1985). Different definitions of prevalence are given in the literature. In the present paper, ‘*proportion of a population that has a disease at a specific point in time*’ is used as a definition (Rothmann and Greenland 1998). In contrast, incidence rate is defined as number of new cases reported per year and per number of persons in the population under continuing practices of detection and reporting. Thus, the first campaign of the new programme of ultrasonography surveys yields the prevalence of thyroid cancer, and subsequent campaigns the incidence rate among people who already participated in the first campaign.

Autopsies of 2,372 of otherwise cancer-free survivors of the atomic bombings of Hiroshima and Nagasaki revealed 106 papillary thyroid microcarcinoma, mostly of the sclerosing variant (Hayashi et al. 2010). This corresponds to a prevalence of 4.5 %. The autopsy results apply mainly to adults. No conclusive data on occult thyroid cancer in children are available. The number of occult carcinomas detected by ultrasonography depends on the equipment and study protocol used. Several other factors like the age–gender structure of the cohort and the country-specific or even group-specific thyroid cancer frequency influence prevalence (first screening) and incidence rate (subsequent screenings) in ultrasonography surveys.

As of 31 July 2013, ultrasonography has been performed on 11 March 2011 for 41,296 children, adolescents and young adults living in thirteen municipalities of the Fukushima Prefecture that were targeted for screening before April 2012 (Fukushima Medical University 2013a). A secondary examination was required for 214 persons with thyroid nodules larger than 5 mm or cysts larger than 20 mm. These examinations were completed for 165 persons allowing a first estimation of thyroid cancer prevalence in the young population. Cytology of fine needle aspiration (FNA) biopsies revealed 14 cases of suspected malignancy. Surgery has been performed for 10 of them; 9 were identified as papillary carcinoma, one as a benign tumour. The 13 cases of confirmed (9) or still suspected (4) thyroid cancer correspond to a prevalence of 13/41,296 = 0.031 %. The prevalence is expected to increase because of possible thyroid cancer cases among the 49 persons for whom secondary examination was required but not completed by 31 July 2013.

In a second group of municipalities that were targeted for screening between April 2012 and March 2013, 135,586 people were screened and 30 cases of suspected thyroid cancer were detected by cytology of FNA biopsies. Only 55 % of the required secondary examinations were already finished by 31 July 2013; thus, the prevalence in these municipalities is expected to increase beyond the present value of 0.022 %.

Ultrasonography has been performed for the UkrAm cohort consisting of Ukrainians who were 18 years old or younger on 26 April 1986, the date of the Chernobyl accident (Tronko et al. 2006). Concerning sex–age distribution of the cohort at the time of the accident and baseline incidence rate (National Cancer Register of Ukraine 2013), the cohort is comparable with the study group in the Fukushima Prefecture. However, the first screening was performed from 1998 to 2000, i.e. 12–14 years after exposure. The study protocol selected nodules sized from 5 to 10 mm for secondary examination only, if further criteria (such as hypoechoic nodule, or nodules with microcalcifications, having irregular contour or extension through the thyroid capsule, or suspicious lymph nodes) were fulfilled (O’Kane et al. 2010). Thus, this protocol leads under otherwise same conditions to a smaller prevalence than the study protocol in the Fukushima Prefecture. Nevertheless, prevalence of confirmed thyroid cancer cases in the UkrAm cohort, which were assessed to be not related to the exposure from the Chernobyl accident, was higher than what is expected for the Fukushima Prefecture, because cohort members at the time of the first screening were older, in average by about 12 years.

Repeated ultrasonography was also performed for the UkrAm cohort (Brenner et al. 2011). Comparison with the baseline thyroid cancer incidence rate in Ukraine allows an assessment of the impact of the screening on reported incidence rates. The effect of an increased surveillance of the thyroid was also analysed for Belarusian and Ukrainian population groups (Jacob et al. 2006a; Likhtarov et al. 2006). Based on these three studies, in the present paper, the baseline (not related to the radiation exposure due to the accident at the Fukushima Dai-ichi NPS) thyroid cancer incidence rate in the Fukushima Prefecture under the conditions of ongoing surveys is estimated.

Long-term thyroid cancer risk due to exposure to external ionizing radiation has been analysed in a number of studies (Ron et al. 1995; Furukawa et al. 2013; Veiga et al. 2012). However, higher thyroid doses among inhabitants of the Fukushima Prefecture were mainly caused by incorporation of radioiodine (WHO 2012). For young age at exposure to radioiodine and the first 20 years after the Chernobyl accident, an increased risk has been demonstrated (Tronko et al. 2006; Brenner et al. 2011; Cardis et al. 2005; Kopecky et al. 2006; Jacob et al. 2006b). However, the risk information is less complete than for external exposures. In the present study, radiation risks from data of the atomic bomb survivors are derived and the consistency with results obtained in studies on people exposed to ^131^I from the Chernobyl nuclear power plant is investigated. The LSS risk function is used to predict excess rates in the Fukushima Prefecture by applying a screening factor that is based on experiences in the UkrAm cohort and differences in the study protocol of the ultrasonography surveys.

## Materials and methods

### Study groups, screening intervals and thyroid dose

The Fukushima Health Management Survey includes ultrasonography of the thyroid of all residents of the Fukushima Prefecture aged up to 18 years on 11 March 2011 (Fukushima Medical University 2013a). Based on post-Chernobyl studies, we calculated thyroid cancer prevalence for two campaigns of the first screening, October 2011 to March 2012, and April 2012 to March 2013 (see Supporting Information for sex–age distributions). Prevalence is also calculated for the sex–age distribution in the ultrasonography survey of children and adolescents in the three non-contaminated prefectures of Aomori, Yamanashi und Nagasaki (Taniguchi et al. 2013).

According to WHO (2013), in one scenario, an average thyroid dose of 50 mSv for the most exposed fraction (a few ten thousand persons) of the surveyed population in the Fukushima Prefecture is assumed here. Since measurements of the iodine content indicate lower doses (Kamada et al. 2012; Kim et al. 2013; Matsuda et al. 2013; Tokonami et al. 2012), however, in a second scenario, an average thyroid dose of 20 mSv was also assumed. Note that individual thyroid doses could have been considerably larger or smaller. For a linear dose response, however, excess incidence rate is determined by the average dose and not by other parameters of the dose distribution.

### Thyroid cancer prevalence detected by ultrasonography

The UkrAm cohort consists of 13,127 Ukrainians who were up to 18 years old on 26 April 1986, the date of the Chernobyl accident (Tronko et al. 2006). During the time of the first screening in the UkrAm cohort, the age-standardized thyroid cancer incidence rate in Ukraine was 1.2 and 4.9 cases per 10^5^ person-years for males and females, respectively (National Cancer Registry of Ukraine 2013). These rates are comparable with those in Japan in 2007 of 2.2 and 7.9 cases per 10^5^ person-years (National Cancer Center 2012). The first round of ultrasonography in the UkrAm cohort was performed 12–14 years after the accident, and 45 pathologically confirmed thyroid cancer cases were detected. Tronko et al. estimated that 11.2 (95 % CI 3.2; 22.5) of these cases would have been detected in the absence of the exposure due to the Chernobyl accident. This corresponds to a prevalence, *P*_UA_, of about 0.09 (95 % CI 0.02; 0.17) %.

In the present paper, the prevalence for the population in Fukushima Prefecture, *P*_Fp_, is estimated by taking into account differences in study protocols and sex–age structures of the screened populations (Eq. 1):

1 $$P_{\text{Fp}} = f_{\text{sp}} P_{\text{UA}} \lambda_{\text{Japan,Fp}} /\lambda_{\text{Ukraine,U1}}$$

where *λ*_Japan,Fp_ and *λ*_Ukraine,U1_ are the average incidence rates in a hypothetical group of Japanese (National Cancer Center 2012) with the sex–age structure of the surveyed Fukushima population and of a hypothetical group of Ukrainians (Fedorenko et al. 2002) with the sex–age structure of the UkrAm cohort during the first survey, respectively. The factor *f*_sp_ accounts for differences in the study protocols in the UkrAm study and the Fukushima survey. The ratio of the numbers of all tumours larger than 5 mm (study protocol in the Fukushima Prefecture) and larger than 10 mm (UkrAm cohort) under otherwise the same conditions is the maximum of *f*_sp_, because also some nodules in the size range from 5 to 10 mm were selected in the UkrAm for further investigation and turned out to be cancer (O’Kane et al. 2010).

No direct information is available to determine *f*_sp_ for tumours. As a surrogate, the corresponding ratio for nodules is used instead. This choice is supported by the thyroid screening study in Hong Kong (Yuen et al. 2011), in which the ratio for nodules larger than 5 mm and larger than 10 mm is 2.4 (398/169). For tumours, the ratio is 2.2 (11/5), nearly equal to that for nodules. The screenings from October 2011 to March 2013 in the Fukushima Prefecture detected 1,125 nodules larger than 5 mm and 354 nodules larger than 10 mm (Fukushima Medical University 2013b). Correspondingly, the upper boundary of *f*_sp_ is estimated to be 1,125/354 = 3.2. The lower boundary is assumed to correspond to no additional cases according to differences in the study protocol, 1.0. The factor *f*_sp_ is assumed to have a symmetrical triangular distribution between these two boundaries with a maximum at 2.1.

### Impact of ultrasonography and thyroid surveillance on thyroid cancer incidence rate

The impact of the second to fourth screenings on the incidence rate in the UkrAm cohort can be calculated as the ratio of the baseline incidence rate in the cohort (Brenner et al. 2011) and the incidence rate in Ukraine in the period 2001–2007 for a hypothetical population with the same sex–age distribution as that of the UkrAm cohort (National Cancer Registry of Ukraine 2013). The baseline incidence rate in the cohort can be estimated by the ratio of the excess absolute rate per unit dose, *EAR*_UA_, and the excess relative risk per unit dose, *ERR*_UA_. Brenner et al. (2011) estimated the *EAR*_UA_ to be 22.1 (95 % CI 0.04; 5.78) cases per 10^5^ person-years per Gy, and the *ERR*_UA_ to 1.91 (95 % CI 0.43; 6.34) Gy^−1^. These numbers indicate a best estimate of the baseline incidence rate in the UkrAm cohort during the second to fourth screening of 11.6 cases per 10^5^ person-years. The incidence rate in Ukraine with the same sex–age structure as in the UkrAm cohort during the second to fourth screening, *λ*_Ukraine,U2–4_, is 3.3 cases per 10^5^ person-years (National Cancer Center of Ukraine 2013). The screening factor in the UkrAm, *f*_UA_, is calculated by

2 $$f_{\text{UA}} = EAR_{\text{UA}} /(ERR_{\text{UA}} \times \lambda_{{{\text{Ukraine}},U2 - 4}} )$$

*EAR*_UA_ and *ERR*_UA_ are correlated, and the coefficient of determination (square of correlation coefficient) has been assumed to be uniformly distributed from 0.7 to 1.0.

Thus, the distribution of the screening factor in the Fukushima Prefecture, *F*_scr_, is a product of the screening factor in the UkrAm cohort, *f*_UA_, and the factor taking into account differences in the study protocol, *f*_sp_.

### Thyroid cancer risk in LSS

Recently, thyroid cancer risk in the cohort of survivors of the atomic bombings of Hiroshima and Nagasaki has been analysed by Furukawa et al. (2013) in the frame of the so-called Life Span Study, (LSS). Their analysis excluded microcarcinoma (tumours smaller than 10 mm). However, tumours smaller than 10 mm are expected to contribute significantly to thyroid cancer prevalence and incidence rate in the Fukushima Prefecture under the conditions of ultrasonography surveys. An earlier analysis of thyroid cancer in the LSS by Preston et al. (2007) included microcarcinoma, but did not provide separate risks for LSS members participating or not participating in screening in the adult health study (AHS) where participants are medically examined every second year. In order to take into account both effects, i.e. those of carcinoma of size smaller than 10 mm and those of increased thyroid surveillance in the AHS, we re-analysed the LSS data. In an excess relative risk model, standard dependences on age-at-exposure, *e*, and attained age, *a*, were applied for those not participating in the AHS programme, while for LSS members participating in the AHS, the baseline thyroid cancer incidence rate was modified by a time-dependent screening factor *F*_AHS_(*a,e*), having different values before and after 1970:

3 $$F_{\text{AHS}} (a,e) = \left\{ {\begin{array}{*{20}c} {\exp (\beta_{\text{AHS,1}} ),} \hfill & {{\text{if }}a - e \ge 25{\text{ (member of AHS in 1970 and later)}}} \hfill \\ {\exp (\beta_{\text{AHS,1}} + \beta_{\text{AHS,2}} ),} \hfill & {\text{otherwise (member of AHS before 1970)}} \hfill \\ \end{array} } \right.$$

These two time periods were chosen to differentiate the early period with many autopsies from the later period with fewer autopsies (Preston et al. 2007). More details on the risk models and their parameters are given in the Electronic Supplementary Material.

### Excess absolute rate in the Fukushima Prefecture

The standard approach of transferring risk estimates for thyroid cancer in the LSS to other populations is assuming a multiplicative interaction of radiation and other risk factors, i.e. the excess relative risk per unit dose (*ERR*) is assumed to be the same in both populations (US Environmental Protection Agency 2011; National Research Council 2006). Thus, the excess absolute rate per unit dose in the population of interest, *EAR*, is obtained by multiplication of *ERR* with the country-specific incidence rate. For the present study, three additional factors were applied: *F*_scr_ (see above), *F*_L_(*a*–*e*) that takes into account that radiation-induced cases are not expected before 3 years after the exposure (Heidenreich et al. 1999) and *F*_DREF_ for the additional uncertainty of the risk function at low dose rate (Jacob et al. 2009). In summary, we calculate in the model of multiplicative interaction

4 $$EAR\left( {s,e,a} \right) = F_{\text{scr}} F_{\text{L}} \left( {a - e} \right)F_{\text{DREF}} ERR_{\text{LSS}} \left( {s,e,a} \right)\lambda_{\text{Japan}} \left( {s,a} \right),$$

where *ERR*_LSS_(*s,e*,*a*) is the excess relative risk per unit dose for LSS cohort members not participating in the AHS, where *s* is an indicator for sex.

Baseline thyroid cancer incidence rate in Japan in 2007 is lower than 10^−6^ per year for males of age younger than 15 years, which is reported as zero in the Japanese cancer centre (National Cancer Center 2012). Based on these data and according to Eq. (4), no cases would be expected during the first 15 years among males exposed as infants. In order to avoid this artefact, an equal probability of any risk between results obtained with models of additive and multiplicative interactions (mixed transfer) is assumed:

5 $$EAR\left( {s,e,a} \right) = F_{\text{scr}} F_{\text{L}} \left( {a - e} \right)F_{\text{DREF}} ERR_{\text{LSS}} \left( {s,e,a} \right)\left[ {f\lambda_{\text{Japan}} \left( {s,a} \right) + \left( { 1- f} \right)\lambda_{\text{LSS}} \left( {s,e,a} \right)} \right]$$

where *f* is assumed to be uniformly distributed between 0 and 1. Similarly, WHO (2013) used weighted average of multiplicative and additive risk transfer, assuming equal weights.

### Attributable risk

The risk rate attributable to radiation exposure, *ARR*, is calculated in Eq. 6 by multiplication of *EAR*(*s,e*,*a*) with thyroid dose, *D*, and taking into account cancer-free survival, *S*(*s,a*), in Japan in 2007 (National Cancer Center 2012; Ministry of Health, Labour and Welfare of Japan 2013; see also Electronic Supplementary Material):

6 $$ARR\left( {s,e,a,D} \right) = EAR\left( {s,e,a} \right) \; D \; S\left( {s,a} \right)/S\left( {s,e} \right)$$

Attributable risks are then obtained by integration over pre-defined periods after exposure:

7 $$AR(s,e,a,D) = \int\limits_{e}^{a} {ARR(s,e,t,D){\text{d}}t}$$

Lifetime attributable risk refers to an integration period from exposure over the whole lifetime.

Similarly, baseline risk is modelled as follows (Eq. 8):

8 $$BR(s,e,a) = F_{\text{scr}}\int\limits_{e}^{a} {\lambda_{\text{Japan}} (s,t)S(s,t){\text{d}}t/S(s,e).}$$

Risks for the study group are calculated by averaging over the sex–age distribution at the time of exposure.

## Results

### Thyroid cancer prevalence

Our estimate of prevalence of pathologically confirmed thyroid cancer cases among the screened population of the municipalities in the Fukushima Prefecture targeted for the survey for the period October 2011 to March 2012 is 0.027 % (95 % CI 0.010 %; 0.050) (Table 1). The large uncertainty of this estimation is mainly caused by the uncertainties of the prevalence of baseline cases in the UkrAm cohort, *P*_UA_, and of the impact of differences of the study protocol, expressed by the factor, *f*_sp_.

**Table 1:** Expected prevalence of confirmed thyroid cancer among screened population groups targeted for the Fukushima Health Management Survey before April 2012, for the period from April 2012 to March 2013, and in the non-contaminated prefectures of Aomori, Yamanashi and Nagasaki (Taniguchi et al. 2013). Hypothetical thyroid cancer incidence rate without screening according to cancer registry data for Japan in 2007 is added to demonstrate the dependence on age

Study group in prefecture(s)	Period of screening	Hypothetical incidence rate without screening (PY^−1^)	Estimated prevalence (%)^a^
Fukushima (2011/2012)	Oct 2011–Mar 2012	0.267 × 10^−5^	0.027 (0.010; 0.050)
Fukushima (2012/2013)	Apr 2012–Mar 2013	0.332 × 10^−5^	0.034 (0.013; 0.061)
Aomori, Yamanashi and Nagasaki	Nov 2012–Jan 2013	0.317 × 10^−5^	0.032 (0.012; 0.057)

*PY* person-years

^a^Arithmetic mean and 95 % confidence interval

Prevalence for the municipalities of the Fukushima Prefecture targeted for later periods is predicted to be somewhat higher, because the screening has been performed later; thus, mean age in the cohort at the time of screening and, correspondingly, average hypothetical incidence rate without screening are higher (see Table 1).

### Screening factors due to ultrasonography surveys

**Fig. 1:**
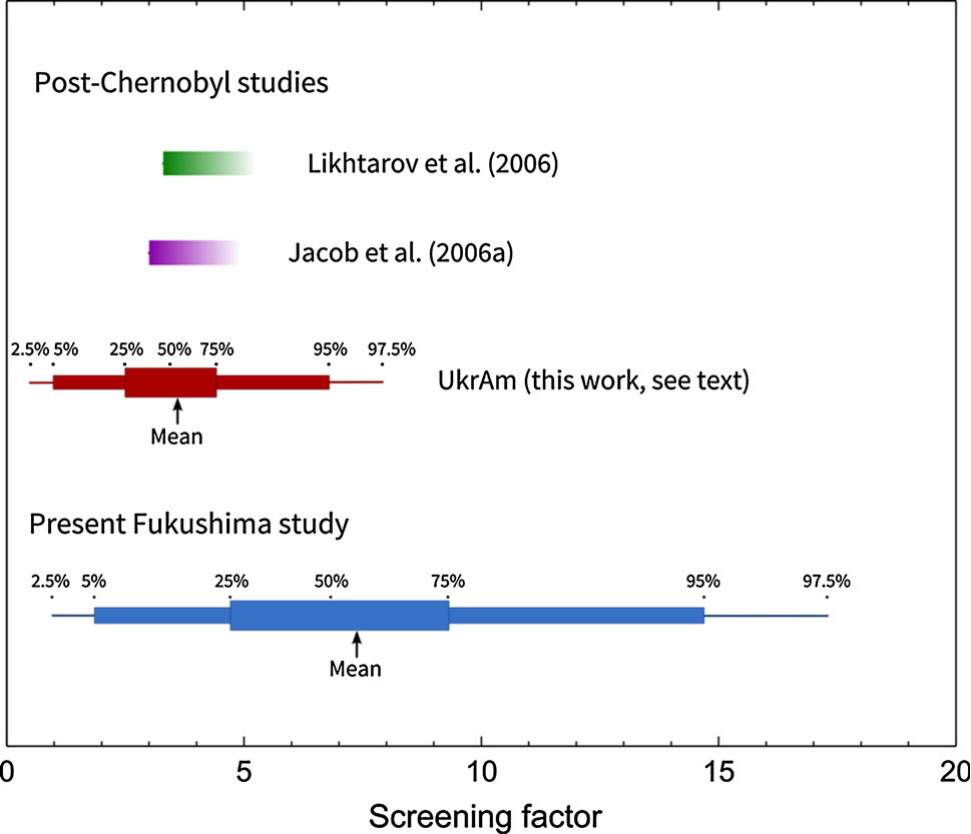
Increase in thyroid cancer incidence rate due to ultrasonography screening. The upper two studies relate to populations without systematic screening; thus, the estimates of the effect of ‘grey’ screening on these populations (*left border of the boxes*) are lower boundaries for the effect of a systematic screening. The screening factor for the Fukushima Prefecture, *F*_scr_, is the product of the screening factor in the UkrAm cohort, *f*_UA_, and a factor taking into account differences in the study protocol, *f*_sp_

Thyroid cancer incidence rate in the UkrAm cohort during the second to fourth screening is estimated to be higher than the national incidence rate in Ukraine by a factor, *f*_UA_, of 3.6, with a 95 % confidence range from 0.5 to 7.9 (Fig. 1).

Thyroid cancer incidence rate in the Fukushima Prefecture under the condition of continued ultrasonography surveys is estimated to be increased compared with the incidence rate in 2007 by a factor of 7.4, with a 95 % confidence range from 0.96 to 17.3. Because of differences in the study protocol, the screening factor in the Fukushima Prefecture is larger and has a wider confidence interval than that in the UkrAm cohort.

### Thyroid cancer incidence risk in the LSS

The impact of screening and autopsies on thyroid cancer incidence among AHS participants was relatively small. For the period before 1970 with a relatively high rate of autopsies, we find a screening factor of 1.72 (95 % CI 1.17; 2.55), and for the period afterwards of 1.23 (95 % CI 0.96; 1.59).

**Fig. 2:**
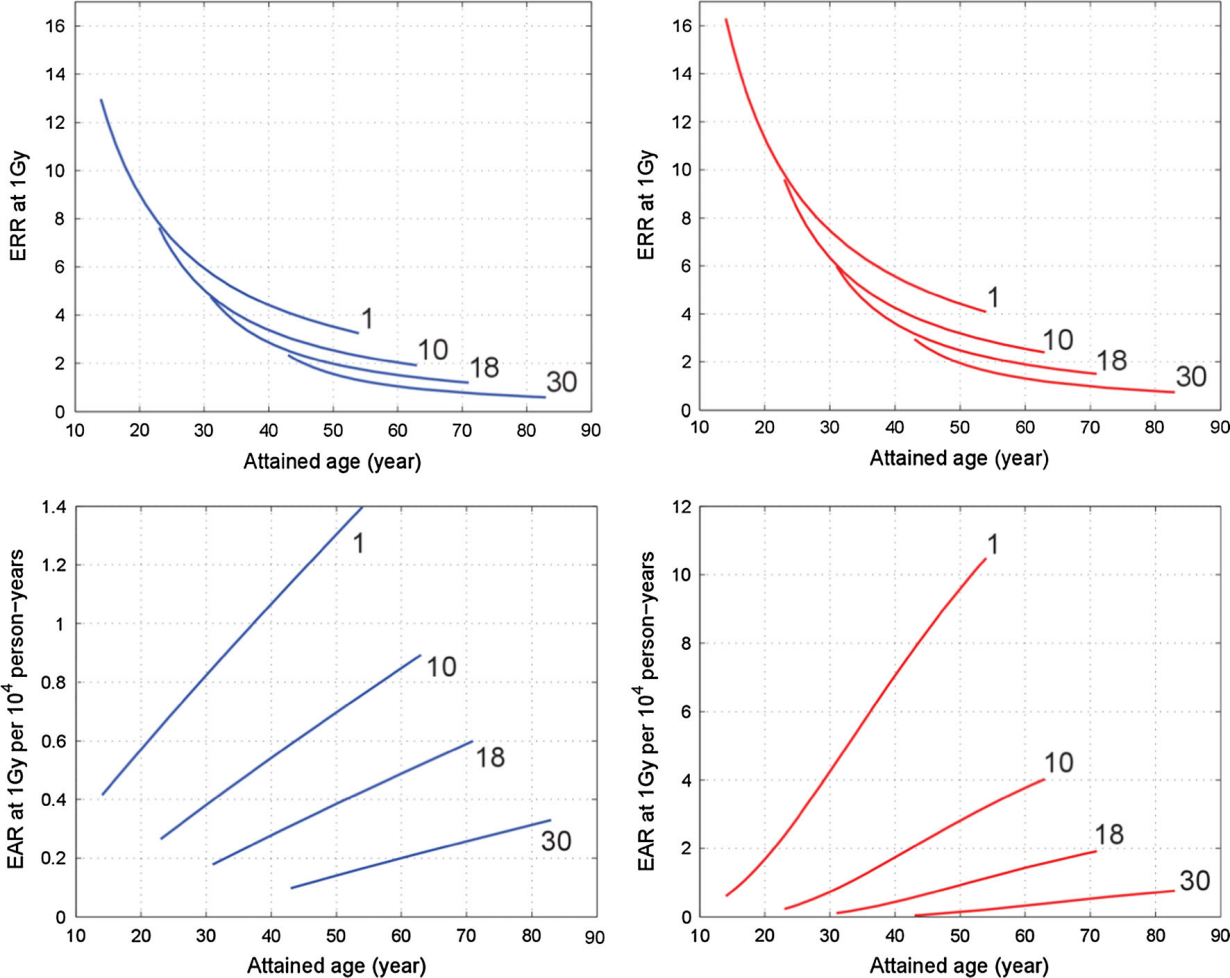
Excess relative risk (*upper panels*) and excess absolute rate (*lower panels*) of thyroid cancer of male (*left panels*, *blue curves*) and female (*right panels*, *red curves*) LSS members not participating in the AHS for different ages at exposure (shown as numbers in years near the curves)

ERR in the LSS decreases with age at exposure and age attained (see Fig. 2). EAR decreases with age at exposure as well. However, it increases with time since exposure.

### Attributable risk rate

**Fig. 3:**
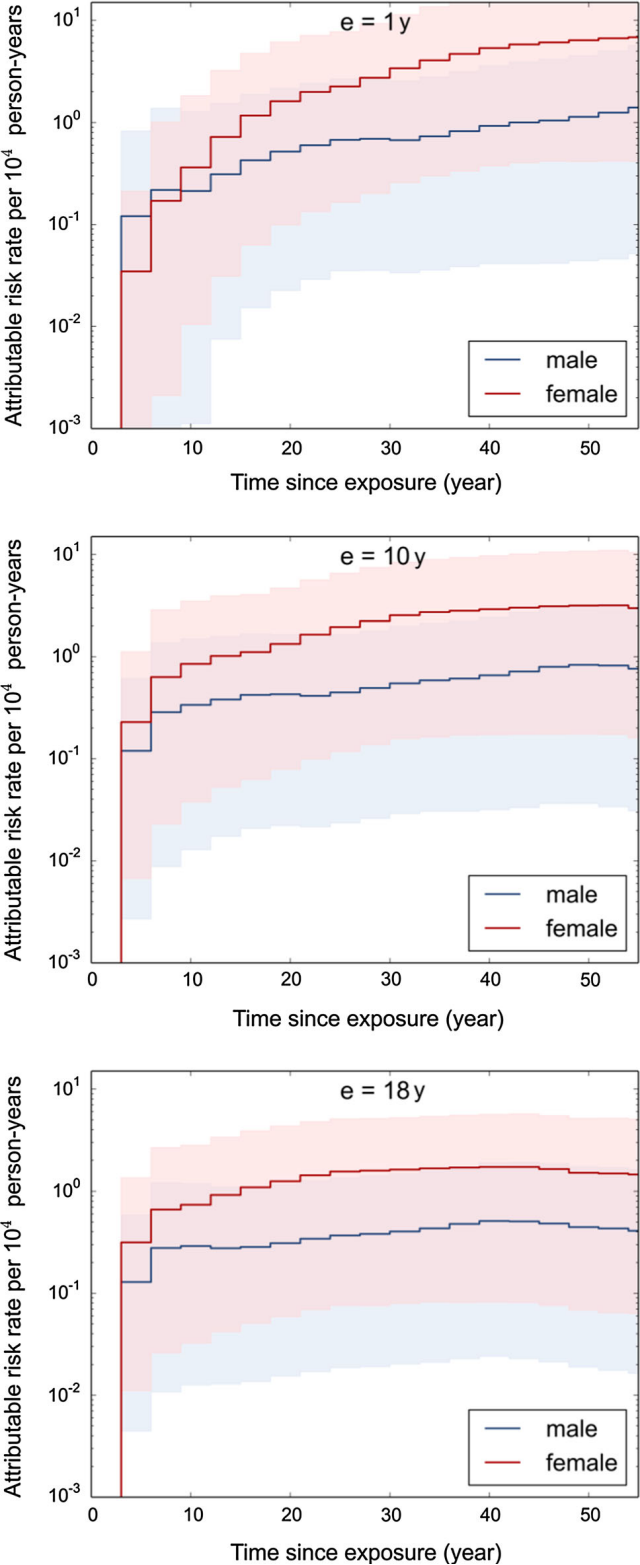
Mixed transfer model for the attributable risk rate of thyroid cancer after exposure at age of 1, 10, and 18 years with a thyroid dose of 100 mGy. Mean values and 95 % confidence intervals are shown

In the mixed transfer model, the risk rate attributable to radiation exposure increases for young ages at exposure after the minimal latency period of 3 years for a few years steeply with time since exposure (Fig. 3), while it continues to increase with a smaller slope for the next 50 years. For example, for an exposure of females at age of 1 year with a thyroid dose of 100 mSv, the attributable risk rate is predicted to increase from about 0.2 cases per 10^4^ person-years at an attained age of 10 years to 6 cases per 10^4^ person-years at an attained age 50 years.

For exposure at older age, the increase is less steep. The attributable risk rate reaches a flat maximum at the attained age of about 60 years and decreases subsequently due to the decrease in the survival function.

In general, best estimates of the attributable risk rate for females are larger than those for males. The ratio is maximal for an attained age of about 40 years with a value of 5.5 for age at exposure of 1 year. The difference is, however, not significant due to the large uncertainty of the estimates. Main sources of uncertainty are the risk function derived from the LSS, the screening factor, and extrapolation of the risk function to low dose rates (in the order of decreasing importance).

With the exception of young attained age, the multiplicative transfer, Eq. (4), gives results very similar to the mixed transfer. For boys below attained age of 15 years and girls below age of 10, the multiplicative transfer results in no excess cases, whereas the mixed transfer gives non-zero results.

### Attributable risk

Most of the long-term attributable risk accumulates over several decades, whereas the risk during the first years after exposure is small (Table 2). The contribution of the first 10 years to the risk over 50 years decreases with increasing age at exposure. The long latency is especially expressed for females: for age at exposure of 1 year, less than 1 % of the attributable risk cumulated during 50 years after exposure (for 100 mSv: 1.4 %) is contributed by the first 10 years (0.013 %).

**Table 2:** Baseline and radiation-attributable thyroid cancer risk (mean value and 95 % confidence interval) after an exposure with a hypothetical thyroid dose of 100 mSv for different time periods after exposure

Age at exposure (years)	Sex	Thyroid cancer	Thyroid cancer risk (%) for different periods after exposure with a thyroid dose of 100 mSv		
			10 years	20 years	50 years
1	Male	Baseline	0.0 (0.0; 0.0)	0.026 (0.003; 0.065)	0.52 (0.06; 1.2)
		Attributable	0.014 (<10^−4^; 0.090)	0.054 (0.002; 0.26)	0.30 (0.015; 1.2)
	Female	Baseline	0.0029 (2 × 10^−4^; 0.0088)	0.089 (0.012; 0.22)	2.3 (0.31; 5.5)
		Attributable	0.013 (3 × 10^−4^; 0.069)	0.12 (0.007; 0.51)	1.4 (0.11; 4.6)
10	Male	Baseline	0.021 (0.003; 0.053)	0.11 (0.015; 0.27)	0.91 (0.12; 2.2)
		Attributable	0.019 (6 × 10^−4^; 0.088)	0.059 (0.003; 0.24)	0.24 (0.013; 0.87)
	Female	Baseline	0.071 (0.008; 0.18)	0.33 (0.04; 0.80)	3.6 (0.44; 8.7)
		Attributable	0.042 (0.002; 0.19)	0.16 (0.008; 0.59)	0.94 (0.058; 3.1)
18	Male	Baseline	0.080 (0.010; 0.20)	0.20 (0.026; 0.50)	1.2 (0.16; 3.0)
		Attributable	0.018 (7 × 10^−4^; 0.077)	0.047 (0.002; 0.19)	0.18 (0.009; 0.64)
	Female	Baseline	0.22 (0.026; 0.52)	0.79 (0.091; 1.8)	4.7 (0.54; 11)
		Attributable	0.044 (0.002; 0.17)	0.15 (0.007; 0.55)	0.63 (0.03; 2.0)

For the same thyroid dose, the attributable risk accumulating over several decades after exposure decreases with increasing age at exposure. However, the effect is relatively modest (about a factor of two for ages at exposure of 1 and 18 years).

Under the condition of continued screening, baseline thyroid cancer risk during the first 50 years after exposure in the screened population of the Fukushima Prefecture is predicted to be 2.2 (95 % CI 0.27; 5.3) % (Table 3). For an average thyroid dose of 20 mSv, the risk related to the radiation exposure amounts to 0.13 (95 % CI 0.005; 0.40) %. Less than 5 % of the radiation-related risk accumulates during the first 10 years after the exposure.

**Table 3:** Predicted thyroid cancer risk (mean and 95 % confidence interval) in the Fukushima Prefecture: baseline and attributable to radiation exposure for thyroid doses of 50 and 20 mSv assumed for the most exposed population groups

Average thyroid dose (mSv)	Thyroid cancer	Thyroid cancer risk (%) for different periods after exposure		
		10 years	20 years	50 years
–	Baseline	0.055 (0.006; 0.14)	0.23 (0.027; 0.58)	2.2 (0.27; 5.3)
20	Attributable	0.0057 (0.0002; 0.025)	0.021 (0.0007; 0.081)	0.13 (0.005; 0.40)
50	Attributable	0.014 (0.0004; 0.063)	0.053 (0.002; 0.20)	0.32 (0.011; 1.0)

## Discussion

### Thyroid cancer prevalence

The first ultrasonography survey in the Fukushima Prefecture is intended to be finished at 31 Mar 2014, about 3 years after the accident at Fukushima Dai-ichi NPS. After the Chernobyl accident, an excess of thyroid cancer cases was not observed before 3 years after exposure (Heidenreich et al. 1999). Thus, the prevalent cases in the Fukushima Prefecture are not assumed to be related to radiation exposure.

As of 31 July 2013, surgery of the thyroid has been performed for ten persons out of 41,296 who lived at the time of the accident in 13 municipalities that were targeted for ultrasonography survey before April 2012 (Fukushima Medical University 2013a). Nine of the cases were papillary carcinoma, one turned out to be a benign nodule. This corresponds to a prevalence of confirmed cases of 9/41,296 = 0.022 %. This number is a lower boundary for the prevalence because

- four persons having suspected malignancy according to cytology of FNA biopsies had not been operated before 31 July 2013;
- only 165 out of 214 individuals, for whom secondary examination was required, have finished such an examination.

Thus, the results obtained so far by the survey are in agreement with our calculations that resulted in a prevalence of 0.027 (0.010; 0.050) % (Table 1).

### Screening factors in post-Chernobyl studies

In a population-based study of Likhtarov et al. (2006), population groups with ultrasonography frequency of more than 1.8 % and less than 0.7 % have been compared. They estimated thyroid cancer incidence rate in the former group to be larger by a factor of 3.3 than in the latter. For Ukrainian oblasts with high thyroid exposures due to the Chernobyl accident and for most oblasts in Belarus, baseline thyroid cancer incidence in 1999 compared with 1988 was assessed to be higher by a factor of about three (Jacob et al. 2006a). This effect was attributed to an increased surveillance of the thyroid. The present result of the screening factor in the UkrAm cohort of 3.6 (95 % CI 0.5; 7.9) is consistent with the assessment of the two population-based studies mentioned above.

### Thyroid cancer risk in atomic bomb survivors versus post-Chernobyl studies

**Fig. 4:**
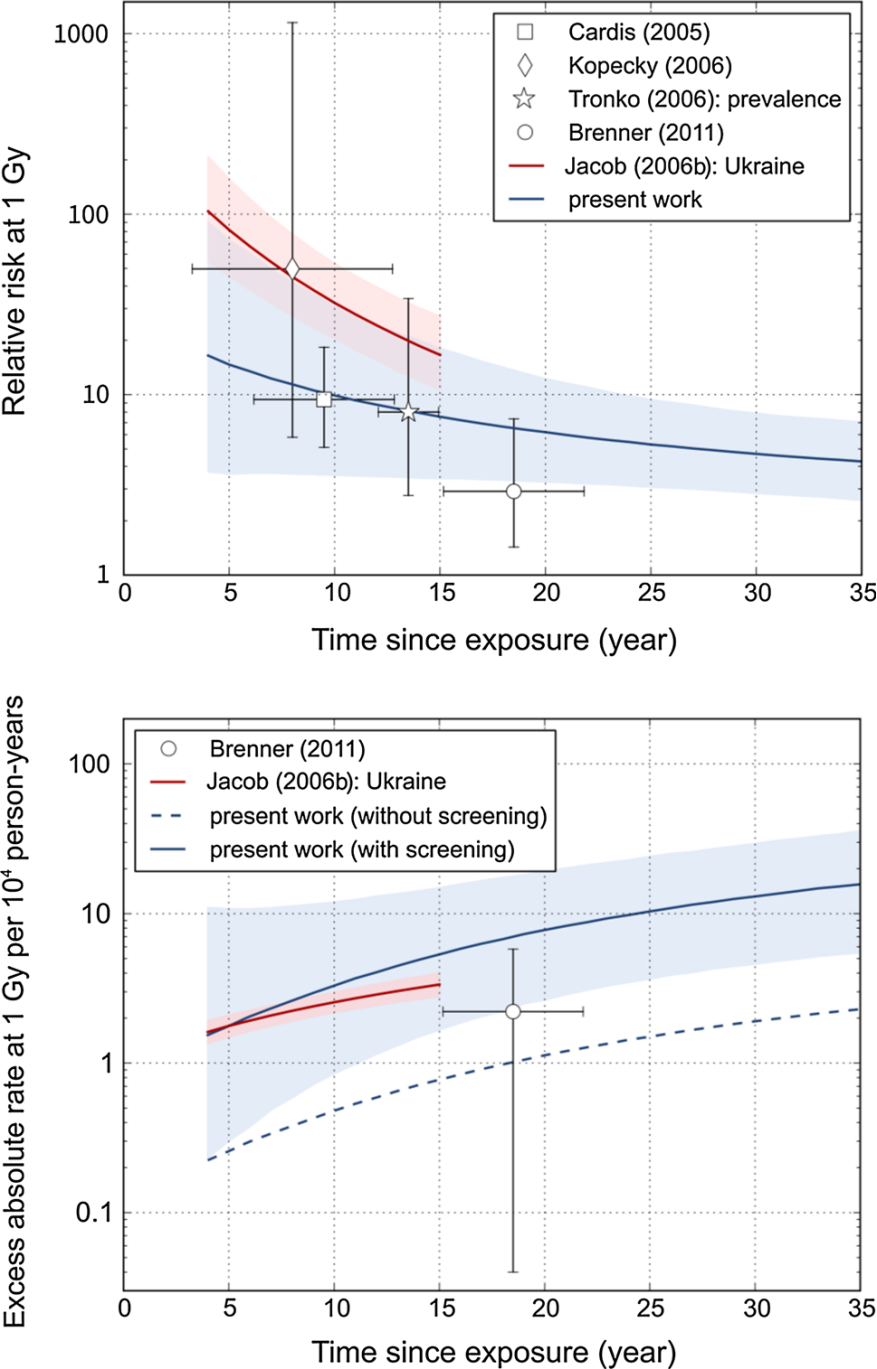
Sex-averaged relative risk (*upper panel*) and excess absolute rate (*lower panel*) of thyroid cancer after an exposure at an age of 7 years (average age at exposure in post-Chernobyl cohort and case–control studies). *Error bars* and *shaded areas* indicate 95 % confidence regions. Results for the LSS members not participating in the AHS with and without the screening factor *F*_scr_ are presented in *blue*, results from post-Chernobyl studies are presented in *red* and by *symbols*

The excess relative risk for thyroid cancer after exposure during childhood with a thyroid dose of 1 Sv in the LSS compares generally well to study results of populations exposed to ^131^I after the Chernobyl accident (Fig. 4, upper panel). For times after exposure of less than 13 years, i.e. when collection of incidence data in the LSS was not yet performed, it cannot be excluded that excess relative risks after exposure at young age are larger than those based on the extrapolation of the LSS function. This would have, however, negligible implications for our results on attributable risk, because the baseline risk in Japan in 2007 for age below 20 years is very small and because there is a good agreement of the excess absolute rate results (see below).

The excess absolute rate depends on the screening conditions. It is relatively small for the LSS members not participating in the AHS (Fig. 4, lower panel, blue dashed line). Applying the full screening factor, *F*_scr_, leads to an EAR estimate (Fig. 4, lower panel, blue solid line) that tends to be higher than what was observed in the UkrAm cohort. This is plausible, because the study protocol in the Fukushima Prefecture is expected to lead to a higher screening effect than in the UkrAm cohort. Indeed, if the screening factor for the UkrAm cohort *f*_UA_ is applied to *EAR*_LSS_, then good agreement with the excess absolute rate per unit dose in the UkrAm cohort is obtained (curve not depicted in Fig. 4).

Overall, these comparisons do not give any evidence against the application of the LSS risk function and the screening factor *F*_scr_ to populations screened in the Fukushima Prefecture.

### Attributable risk rate

In our approach with a mixed transfer of excess risk estimates from the LSS to the population in the Fukushima Prefecture, the attributable risk rate increases steeply after a minimal latency period of 3 years. In contrast, according to the multiplicative transfer, excess cases do not appear before attained age of 15 for males and age attained 10 for females. The latter approach is not in accordance with experiences after the Chernobyl accident, however, where many excess cases were observed before attained age of 10 years (UNSCEAR 2011).

### Attributable risk

According to the results shown in Table 2 for an age of exposure of 1 year, about 15 % of the attributable risk accumulated over 20 years is contributed by the first 10 years after exposure. For an age of exposure of 10 years, we obtain a contribution of 30 %. These predictions compare well to thyroid cancer incidence after the Chernobyl accident. In Belarus, for example, where most of the observed thyroid cancer incidence was attributed to radiation exposure (Jacob et al. 2006a), 29 % of thyroid cancer cases during the first 20 years after exposure occurred during the first 10 years for the age-at-exposure group of 0–4 years, and 35 % for the age-at-exposure group of 5–9 years (UNSCEAR 2011).

Another check of consistency with post-Chernobyl experiences relates to age-at-exposure dependence. According to our prediction, the attributable risk accumulated over the first 20 years after exposure is about the same for ages at exposure of 1 and 10 years, if the thyroid dose is the same. In Belarus, the number of cases was higher for the age-at-exposure group of 0–4 years than for the age-at-exposure group of 5–9 years by a factor of 1.7 (UNSCEAR 2011). Thyroid doses differed by a similar factor (Jacob et al. 2006a).

Finally, we predict the excess rate during the first 20 years after exposure among females to be higher than among males by a factor of 2–3. Again, in Belarus, the risk was higher by a factor of 2.6 (crude rate among females of 6.7 versus 2.6 per 10^4^ person-years for males, according to UNSCEAR (2011)).

Whereas our predictions for the first 20 years after exposure are supported by some studies of thyroid cancer after the Chernobyl accident, our longer-time predictions are more uncertain and may overestimate the risk. This is so because we have neglected a so-called *harvesting effect* that might appear after 20 years after exposure: Early detection may lead to lower numbers of detected cases at later campaigns of a survey. However, we are not aware of studies that give evidence of a harvesting effect of ultrasonography for thyroid cancer incidence.

For a period of 50 years after exposure at ages at exposure of up to 18 years, we predict that an average thyroid dose of 20 mSv would result in a relative contribution of about 6 % to the total thyroid cancer risk. This is in accordance with the results of WHO (2013), because of the close similarity of the approaches applied concerning relative risks. Concerning the total thyroid cancer risk, however, our results are higher by a factor of about 7, because WHO did not consider in its calculations the impact of the ultrasonography survey.

## Conclusion

Based on the results presented here, it is expected that the ultrasonography survey of residents of Fukushima Prefecture will increase thyroid cancer incidence compared with thyroid cancer incidence in 2007 in Japan drastically. The estimated increase has a large uncertainty with a best estimate of a factor of about 7. For an average thyroid dose of 20 mSv of the most exposed in the surveyed population, it is estimated that in the early period, about 10 % and in later periods about 5 % of the reported incidence will be attributable to radiation exposure. Thus, the fraction of thyroid cancer cases attributable to radiation exposure will be small, although there are regional differences due to varying doses. The present assessment includes large uncertainties caused by uncertainties in the thyroid cancer risk function for the LSS, the impact of the ultrasonography survey, and the transfer of the risk function to low-dose exposures to ^131^I. Independent of these uncertainties, however, the order of magnitude of the predicted thyroid cancer prevalence and incidence rate may help to be prepared for a relatively large number of thyroid cancer cases that are to be expected. It is emphasized that most of the cases would not have become clinically relevant without the ultrasonography survey.

## Electronic supplementary material

Below is the link to the electronic supplementary material.

Supplementary material 1 (DOCX 319 kb)
