# Supplementary material for: Ultrasonography survey and thyroid cancer in the Fukushima Prefecture
Source: Radiat Environ Biophys. 2014 Jan 9;53(2):391–401. doi: 10.1007/s00411-013-0508-3 (PMC3996282; doi:10.1007/s00411-013-0508-3)
Supplement: Supplementary file 1 — Supplementary material 1 (DOCX 319 kb) [file 411_2013_508_MOESM1_ESM.docx]

Electronic Supplementary Material

Ultrasonography surveys and thyroid cancer
after the Fukushima accident

**Peter Jacob, Jan Christian Kaiser, Alexander Ulanovsky**

Helmholtz Zentrum München – German Research Center for Environmental Health,
Department of Radiation Sciences, Institute of Radiation Protection,
Ingolstädter Landstraße 1, D-85764, Neuherberg, Germany

Table of Contents

[1. Data for calculating prevalence 1](#_Toc363655954)

[2. Re-analysis of thyroid cancer incidence in the LSS cohort 2](#_Toc363655955)

[2.1 Data set and the cohort 2](#_Toc363655956)

[2.2 Baseline model 2](#_Toc363655957)

[2.3 Dose response 3](#_Toc363655958)

[3. Excess absolute risk in study groups 7](#_Toc363655959)

[3.1 Modifying factors 7](#_Toc363655960)

[3.2 Ratio of baseline incidence rates in Japan in 2007 and in the LSS (non-participants in the AHS) 7](#_Toc363655961)

[3.3 Probability distributions of excess absolute risk 8](#_Toc363655962)

[4. Cancer-free survival function for Japan in 2007 8](#_Toc363655963)

[5. Parameters of risk function from Jacob et al (2006a) 8](#_Toc363655964)

# 1. Data for calculating prevalence

Age- and gender-structure for children and adolescents in Fukushima prefecture have been taken from the data of Fukushima Medical University (2013), and for non-contaminated areas in prefectures Aomori, Yamanashi and Nagasaki from the recent study by (Taniguchi et al. 2013). The numerical values are shown in Table S1.

Table S 1. Age- and gender-distribution of children and young adults subjected to ultrosonographic screening in Fukushima (Fukushima Medical University 2013) and Aomori, Yamanashi, Nagasaki (Taniguchi et al. 2013) prefectures.

| Age (year) | Fukushima  (November 2011 – March 2012) | | Fukushima  (April 2012 – March 2013) | | Aomori, Yamanashi, and Nagasaki  (November 2012 – January 2013) | |
| --- | --- | --- | --- | --- | --- | --- |
|  | male | female | male | female | male | female |
| 0–5 | 5,386 | 5,249 | 19,164 | 18,072 | 96^a^ | 93^a^ |
| 6–10 | 5,757 | 5,492 | 20,587 | 19,245 | 621 | 654 |
| 11–15 | 6,019 | 5,990 | 20,381 | 20,006 | 1,005 | 990 |
| 16–18 | 3,139 | 3,270 | 7,899 | 8,720 | 353 | 553 |
| ^a^ ages from 3 to 5 years | | | | | | |

The cohort in the UkrAm study in the first screening (2-year-screening was performed in 1998–2000) encountered 6,471 males and 6,656 females with an age distribution spanning from 12 to 33 years (Tronko et al 2006). Thyroid cancer incidence in Ukraine during this time has been taken from the National Cancer Registry of Ukraine (Fedorenko et al. 2002). Using these data, thyroid cancer incidence rate weighted with an age- and sex-distribution specific to the first screening in the UkrAm cohort results in the following value: *λ_Ukraine,U1_*=1.756×10^−5^ per person year (PY).

Thyroid cancer incidence in Japan in 2007 has been taken as reported by Japan National Cancer Center (2012). Using age- and sex-distributions for children and young adults in Fukushima prefecture shown in Table S1, one gets average incidence rates of *λ_Japan,Fp_* =0.267×10^−5^ per PY (screening in 2011–2012) and 0.332×10^−5^ per PY (screening in 2012–2013). For the cohort of children and adolescents from prefectures Aomori, Yamanashi and Nagasaki the incidence rate, weighted according to distributions shown in Table S1, equals to 0.317 ×10^−5^ per PY.

# 2. Re-analysis of thyroid cancer incidence in the LSS cohort

## 2.1 Data set and the cohort

The analysis is based on the LSS cohort data for cancer incidence in 1958–1998 contained in the file lssinc07ahs.csv, which has been downloaded from the RERF website ([http://www.rerf.or.jp](http://www.rerf.or.jp/)). It comprises 105,427 subjects and 471 cases of incidental thyroid cancer recorded in 2,764,725 person years (PYs). The crude data is summarized in Table A18 of Preston et al. (2007). The person-year weighted means are 23 years for age at exposure, 53 years for attained age, 60 years for age of cases and 105 mGy for the weighted dose to the thyroid. In the dose calculation, the neutron-related component was weighted with a relative biological effectiveness (RBE) of 10.

## 2.2 Baseline model

The baseline incidence rate *λ*_0_(*s*, *a*, *e*, *c, AHS*, *NIC*) depends on explanatory variables of sex *s*, attained age *a*, age at exposure *e*, city (Hiroshima: *c*=1; Nagasaki *c*=2), status of AHS participation (no: *AHS*=0; yes: *AHS*=1) and of having been in the city at the time of the bombing (for distance from hypocenter <10 km: *NIC*=0; otherwise: *NIC*=1). The baseline incidence rate factorizes

$$\lambda_{0,LSS}\left(s,a,e,c,AHS,NIC\right)\times10^{4}=\lambda_{0,fit}\left(s,a,e\right) F_{scr}\left(a,e,AHS\right) g\left(c,NIC\right)$$

into a fit function common for all cohort members:

$$\lambda_{0,fit}\left(s,a,e\right)=\exp\left(\beta_{0,s}+\beta_{a_{1},s}\ln\frac{a}{60}+\beta_{a_{2},s}\ln^{2}\frac{a}{60}+\beta_{e_{1},s}\frac{e-20}{10}+\beta_{e_{2},s}\left(\frac{e-20}{60}\right)^{2}\right)$$

and an adjustment factor accounting for screening effect for members AHS:

$F_{scr}\left(a,e,AHS\right)=\exp\left(\beta_{AHS}\left(a-e\right) AHS\right)$,

where for non-zero factor *AHS*:

$$\beta_{AHS}\left(a-e\right)=\left\{\begin{matrix} \beta_{AHS,1}, & a-e\geq25\text{ (AHS in 1970 and later);} \\ \beta_{AHS,1}+\beta_{AHS,2}, & a-e<25\text{ (AHS before 1970).} \end{matrix}\right.$$

and a factor accounting for residential status (city and ‘not-in-the-city’ factor – NIC):

$g\left(c,NIC\right)=\exp\left(\beta_{c}\left(c-1\right)+\beta_{NIC}NIC\right)$.

The baseline model of Preston et al. (2007) is nested to the baseline model of the present analysis. In the further calculations, *city*- and *NIC*-status have been averaged out with weights defined from the number of cancer cases observed in each of the sub-groups of the LSS cohort.

## 2.3 Dose response

For the dose response an excess relative risk (ERR) model was chosen using the form

$$ERR\left(s,e,a\right)=\alpha_{d} D\exp\left(\alpha_{s}s+\alpha_{a}\ln\frac{a}{60}+\alpha_{e}\frac{e-20}{10}\right)$$

where *α*... are parameters, *D* is the weighted thyroid dose and parameter *s* equals to +1 for females and to −1 for males.

The original ERR model of Preston et al (2007) used 22 parameters with a deviance of 3,037.97 (Akaike Information Criterion *AIC*=3,081.97). The present model consumed 17 parameters and yielded a deviance of 3,037.65 (*AIC*=3,069.65). Maximum likelihood estimates (MLE) and confidence intervals are given in Table S2. Estimates of the ERR at 1 Gy from Preston et al (2007) and the present study differ by less than 10 percent. The confidence intervals are quite symmetrical. Table S3 gives standard deviations and the correlation matrix of the parameters.

**Table S2** Maximum likelihood estimates (MLE) and confidence intervals (CIs) for the parameters of the ERR model from the present analysis. CIs are calculated from the likelihood profile.

| Name | Unit | MLE estimate and confidence intervals |
| --- | --- | --- |
| β_0,m_ | – | −0.39 (−0.62; −0.17)^a^ |
| β_0,f_ | – | 0.53 (0.36; 0.70)^a^ |
| β_city_ | – | −0.22 (−0.33; −0.11)^a^ |
| β_AHS_ | – | 0.21 (0.08; 0.33)^a^ |
| β_AHS,1970_ | – | 0.33 (0.22; 0.44)^a^ |
| β_NIC_ | – | −0.47 (−0.61; −0.34)^a^ |
| β_a1,m_ | – | 1.9 (1.3; 2.5)^a^ |
| β_a1,f_ | – | 2.0 (1.5; 2.4)^a^ |
| β_a2,f_ | – | −0.75 (−1.30; −0.24)^a^ |
| β_e1,m_ | yr^−1^ | 0.091 (n.a.; 0.19)^a^ |
| β_e1,f_ | yr^−1^ | −0.24 (−0.33; −0.16)^a^ |
| β_e2,f_ | yr^−2^ | 0.080 (0.060; 0.098)^a^ |
| α_d_ | Gy^−1^ | 1.07 (0.71; 1.51)^a^ (95% 0.44; 2.04)^b^ |
| α_e_ | – | −0.59 (−0.89; −0.32)^a^ (95% −1.20; −0.08)^b^ |
| α_a_ | yr^−1^ | −1.03 (−1.89; −0.16)^a^ (95% −2.74; 0.70)^b^ |
| α_s_ | – | 0.11 (−0.16; 0.42)^a^ (95% −0.52; 0.77)^b^ |

^a^ 68% confidence interval

^b^ 95% confidence interval

**Table S3** Maximum likelihood estimates (MLE), standard deviations and covariance matrix of parameters of the ERR model for thyroid cancer incidence in the LSS. Std.dev – standard deviation

| Parameter | MLE | Std.dev | Covariance matrix | | | | | | | |
| --- | --- | --- | --- | --- | --- | --- | --- | --- | --- | --- |
|  |  |  |  | $\beta_{0,f}$ | $\beta_{\text{city}}$ | $\beta_{\text{ahs},1}$ | $\beta_{\text{ahs},2}$ | $\beta_{\text{NIC}}$ | $\beta_{a_{1},m}$ | $\beta_{a_{1},f}$ |
|  | -3.9E-01 | 2.3E-01 | 5.2E-02 | 2.3E-02 | -3.1E-03 | -1.9E-02 | 5.7E-03 | -2.2E-03 | 5.5E-02 | 1.7E-02 |
| $\beta_{0,f}$ | 5.3E-01 | 1.7E-01 | 2.3E-02 | 2.9E-02 | -3.0E-03 | -1.8E-02 | 5.2E-03 | -2.5E-03 | 1.6E-02 | 1.8E-02 |
| $\beta_{\text{city}}$ | -2.2E-01 | 1.1E-01 | -3.1E-03 | -3.0E-03 | 1.2E-02 | -2.2E-04 | 9.2E-05 | 9.5E-04 | 4.3E-04 | 5.9E-04 |
| $\beta_{\text{ahs},1}$ | 2.1E-01 | 1.3E-01 | -1.9E-02 | -1.8E-02 | -2.2E-04 | 1.6E-02 | -5.5E-03 | -1.5E-03 | -1.2E-02 | -1.4E-02 |
| $\beta_{\text{ahs},2}$ | 3.3E-01 | 1.1E-01 | 5.7E-03 | 5.2E-03 | 9.2E-05 | -5.5E-03 | 1.2E-02 | -8.0E-05 | 2.6E-02 | 3.1E-02 |
| $\beta_{\text{NIC}}$ | -4.7E-01 | 1.3E-01 | -2.2E-03 | -2.5E-03 | 9.5E-04 | -1.5E-03 | -8.0E-05 | 1.8E-02 | -2.6E-04 | 6.2E-04 |
| $\beta_{a_{1},m}$ | 1.9E+00 | 5.9E-01 | 5.5E-02 | 1.6E-02 | 4.3E-04 | -1.2E-02 | 2.6E-02 | -2.6E-04 | 3.5E-01 | 7.9E-02 |
| $\beta_{a_{1},f}$ | 2.0E+00 | 4.4E-01 | 1.7E-02 | 1.8E-02 | 5.9E-04 | -1.4E-02 | 3.1E-02 | 6.2E-04 | 7.9E-02 | 1.9E-01 |
| $\beta_{a_{2},f}$ | -7.5E-01 | 5.3E-01 | -1.1E-03 | -6.9E-03 | 3.4E-04 | -4.6E-03 | 1.3E-02 | -5.4E-05 | -1.3E-02 | 1.1E-01 |
| $\beta_{e_{1},m}$ | 9.1E-02 | 1.0E-01 | -1.4E-02 | -2.5E-03 | 1.8E-04 | 2.1E-03 | -4.8E-03 | 1.2E-04 | -4.5E-02 | -1.4E-02 |
| $\beta_{e_{1},f}$ | -2.4E-01 | 8.3E-02 | -2.5E-03 | -2.9E-03 | 2.3E-04 | 1.5E-03 | -4.5E-03 | 5.9E-05 | -1.3E-02 | -2.4E-02 |
| $\beta_{e2,f}$ | 8.0E-02 | 1.9E-02 | -4.0E-05 | -4.5E-04 | -6.8E-06 | 1.8E-04 | -1.7E-04 | 5.5E-05 | 2.4E-04 | -1.3E-05 |
| $\alpha_{d}$ | 1.1E+00 | 4.0E-01 | -2.7E-03 | 8.4E-03 | 1.1E-03 | -1.8E-02 | 2.3E-03 | 1.1E-02 | 2.1E-03 | 7.6E-04 |
| $\alpha_{e}$ | -5.9E-01 | 2.8E-01 | 1.1E-02 | 5.9E-03 | -3.2E-05 | -4.3E-03 | -5.0E-03 | 3.2E-03 | 9.0E-03 | 9.8E-03 |
| $\alpha_{a}$ | -1.0E+00 | 8.4E-01 | -2.1E-02 | -1.8E-02 | 2.0E-04 | -2.8E-03 | 1.3E-02 | -3.3E-04 | -1.1E-01 | -4.4E-02 |
| $\alpha_{g}$ | 1.1E-01 | 2.8E-01 | 2.0E-02 | -2.4E-03 | 1.6E-04 | -1.5E-03 | -5.2E-04 | 9.4E-04 | -2.5E-02 | 6.7E-03 |

**Table S3 (cont’d).**

| Parameter | Covariance matrix (cont’d) | | | | | | | |
| --- | --- | --- | --- | --- | --- | --- | --- | --- |
|  | $\beta_{a_{2},f}$ | $\beta_{e_{1},m}$ | $\beta_{e_{1},f}$ | $\beta_{e2,f}$ | $\alpha_{d}$ | $\alpha_{e}$ | $\alpha_{a}$ | $\alpha_{g}$ |
|  | -1.1E-03 | -1.4E-02 | -2.5E-03 | -4.0E-05 | -2.7E-03 | 1.1E-02 | -2.1E-02 | 2.0E-02 |
| $\beta_{0,f}$ | -6.9E-03 | -2.5E-03 | -2.9E-03 | -4.5E-04 | 8.4E-03 | 5.9E-03 | -1.8E-02 | -2.4E-03 |
| $\beta_{\text{city}}$ | 3.4E-04 | 1.8E-04 | 2.3E-04 | -6.8E-06 | 1.1E-03 | -3.2E-05 | 2.0E-04 | 1.6E-04 |
| $\beta_{\text{ahs},1}$ | -4.6E-03 | 2.1E-03 | 1.5E-03 | 1.8E-04 | -1.8E-02 | -4.3E-03 | -2.8E-03 | -1.5E-03 |
| $\beta_{\text{ahs},2}$ | 1.3E-02 | -4.8E-03 | -4.5E-03 | -1.7E-04 | 2.3E-03 | -5.0E-03 | 1.3E-02 | -5.2E-04 |
| $\beta_{\text{NIC}}$ | -5.4E-05 | 1.2E-04 | 5.9E-05 | 5.5E-05 | 1.1E-02 | 3.2E-03 | -3.3E-04 | 9.4E-04 |
| $\beta_{a_{1},m}$ | -1.3E-02 | -4.5E-02 | -1.3E-02 | 2.4E-04 | 2.1E-03 | 9.0E-03 | -1.1E-01 | -2.5E-02 |
| $\beta_{a_{1},f}$ | 1.1E-01 | -1.4E-02 | -2.4E-02 | -1.3E-05 | 7.6E-04 | 9.8E-03 | -4.4E-02 | 6.7E-03 |
| $\beta_{a_{2},f}$ | 2.8E-01 | -9.0E-04 | -2.9E-03 | -2.8E-03 | 4.1E-02 | -2.3E-02 | 1.9E-01 | -8.4E-03 |
| $\beta_{e_{1},m}$ | -9.0E-04 | 1.0E-02 | 2.6E-03 | -9.4E-05 | 5.5E-03 | -3.2E-03 | 1.3E-02 | -4.1E-03 |
| $\beta_{e_{1},f}$ | -2.9E-03 | 2.6E-03 | 6.9E-03 | -9.6E-04 | 2.4E-03 | -8.3E-03 | 1.8E-02 | 5.1E-04 |
| $\beta_{e2,f}$ | -2.8E-03 | -9.4E-05 | -9.6E-04 | 3.8E-04 | -2.9E-04 | 2.2E-03 | -4.2E-03 | 7.3E-05 |
| $\alpha_{d}$ | 4.1E-02 | 5.5E-03 | 2.4E-03 | -2.9E-04 | 1.6E-01 | 1.2E-02 | 1.3E-01 | -5.0E-02 |
| $\alpha_{e}$ | -2.3E-02 | -3.2E-03 | -8.3E-03 | 2.2E-03 | 1.2E-02 | 7.7E-02 | -1.4E-01 | 1.1E-02 |
| $\alpha_{a}$ | 1.9E-01 | 1.3E-02 | 1.8E-02 | -4.2E-03 | 1.3E-01 | -1.4E-01 | 7.0E-01 | -2.2E-02 |
| $\alpha_{g}$ | -8.4E-03 | -4.1E-03 | 5.1E-04 | 7.3E-05 | -5.0E-02 | 1.1E-02 | -2.2E-02 | 8.1E-02 |

# 3. Excess absolute risk in study groups

## 3.1 Modifying factors

The onset of radiation-related excess in thyroid cancer incidence has been modelled as dimensionless function of time since exposure[[1]](#footnote-1):

$F_{L}\left(t\right)=\left(1+\exp\left(\frac{4-t}{0.218}\right)\right)^{-1}$.

The uncertainty introduced by extrapolating the LSS-based risk function to low-dose-rate exposures, as they occurred mainly after the Fukushima accident, has been modelled by the corresponding modifying factor *F_DREF_*, which has lognormal distribution with a mean value of 1.0 and a boundaries of the 95 % confidence interval at 0.4 and 2.1.

## 3.2 Ratio of baseline incidence rates in Japan in 2007 and in the LSS (non-participants in the AHS) – Fig. S2

|  |  |
| --- | --- |
| 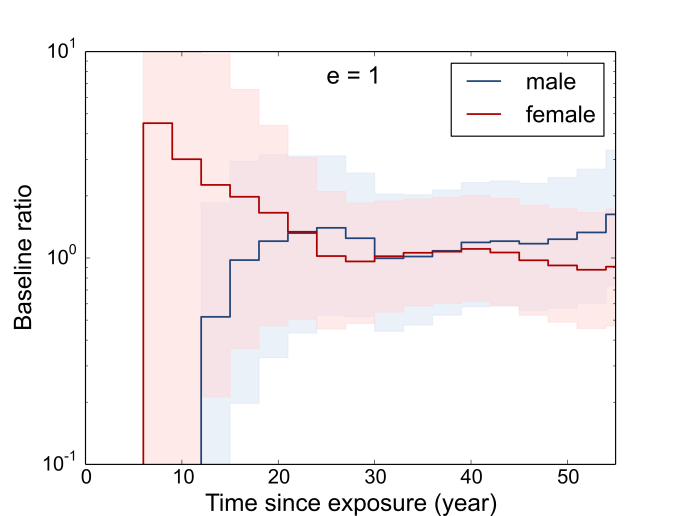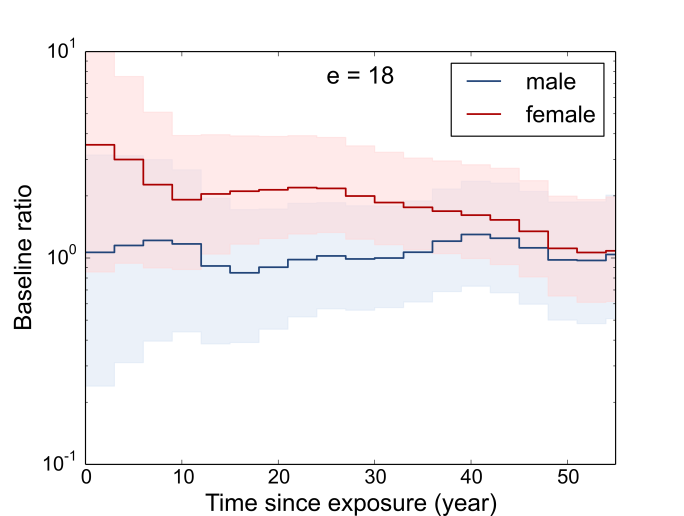 | 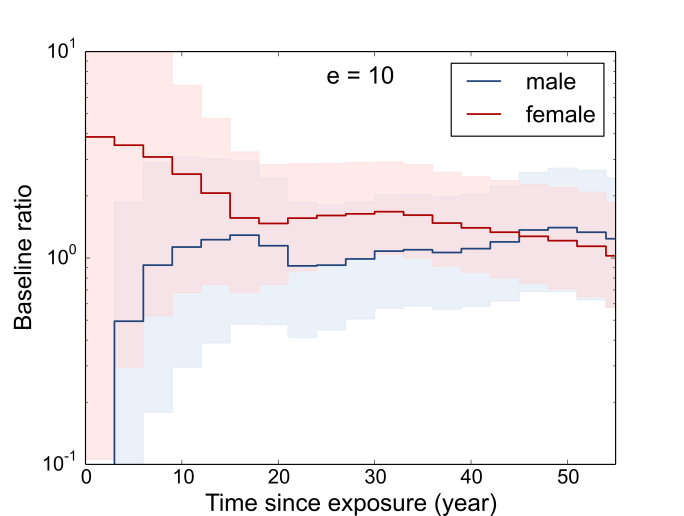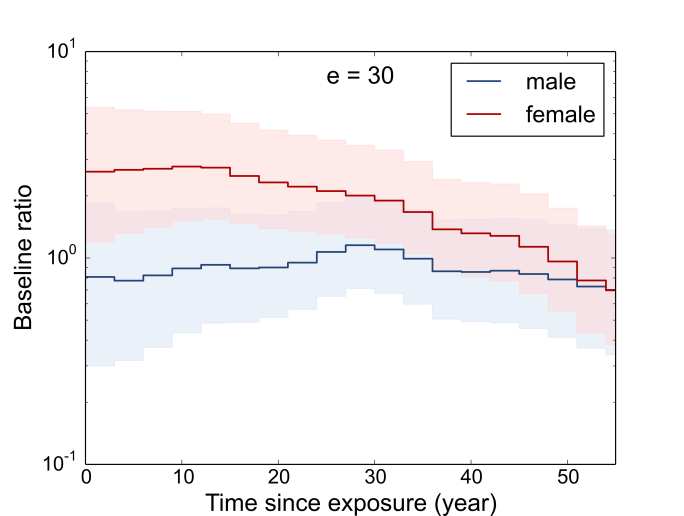 |

**Fig. S2** Ratio of incidence rates for Japan in 2007 and baseline model for LSS members not participating in the AHS.

## 3.3 Probability distributions of excess absolute risk

Probability distributions of risk estimates have been obtained by stochastic simulations. Parameter values have been sampled from:

- multivariable normal distribution using best estimates and covariances given in Table S3;
- uniform distribution of the transfer factor *f* ;
- uncertainty distributions of the modifying factors *F_scr_* and *F_DREF_* (see sub-section 3.1);
- Poisson distribution of the number of observed baseline cases in Japan in the corresponding 5-year age groups.

The risk models are then used to calculate risk estimates for given age at exposure *e*, attained age *a*, and a thyroid dose of 100 mGy. The procedure was repeated 10,000 times to obtain distributions of the risk estimates.

# 4. Cancer-free survival function for Japan in 2007

The survival function, *S*(*a*), is related to the mortality rate $\mu(t)$by:

$S(a)=\exp\left(-\int_{0}^{a} \mu\left(t\right)dt\right)$.

Cancer-free survival function accounts only for those persons who had no prior cancer. This function is computed from all-cause mortality rate $\mu(t)$ modified by all-cancer incidence rate $\lambda_{C}(a)$ and all-cancer mortality rate $\mu_{C}(a)$ at age *a*, thus the cancer-free survival function is computed as follows:

$S^{*}(a)=\exp\left(-\int_{0}^{a} \left(\mu(t)+\lambda_{C}(t)-\mu_{C}(t)\right) dt\right)$.

The survival function has been computed using all-cause mortality rate from Abridged Life Tables for Japan 2007 (Ministry of Health, Labour and Welfare of Japan 2013) and all-cancer incidence rate and all-cancer mortality rate from the data of Japan National Cancer Center (2012).

# 5. Parameters of risk function from Jacob et al (2006a)

Cancer baseline rate and EAR are characterized as functions of attained age *a*, age at exposure *e*, and thyroid dose *d*:

$\lambda_{0}=\exp\left(\beta_{0}+\beta_{c}F_{c}+\beta_{s}F_{s}+\beta_{a}\ln\frac{a}{20}+\beta_{e}\left(e-10\right)\right)$,

$$EAR=\left(\alpha_{d_{1}}d+\alpha_{d_{2}}d^{2}\right)\exp\left(\alpha_{c}F_{c}+\alpha_{s}F_{s}+\alpha_{a}\ln\frac{a}{20}+\alpha_{e}\left(e-10\right)\right)$$

where country- and gender-dependent factors are:

$F_{c}=\left\{\begin{matrix} -0.5, & \text{Ukraine} \\ +0.5, & \text{Belarus} \end{matrix}\right.$ and $F_{s}=\left\{\begin{matrix} -0.5, & \text{male} \\ +0.5, & \text{female} \end{matrix}\right.$

and values of model parameters and their covariance matrix are given in Table S4.

**Table S4** Parameters of the EAR model for thyroid cancer according to Jacob et al (2006a):
Maximum likelihood estimates (MLEs), standard deviations (std.dev.)and covariance matrix (own calculation).

| Parameter | MLE | Std.dev. | Covariance matrix | | | | | | | | | |
| --- | --- | --- | --- | --- | --- | --- | --- | --- | --- | --- | --- | --- |
|  |  |  | $\beta_{0}$ | $\beta_{c}$ | $\beta_{s}$ | $\beta_{a}$ | $\beta_{e}$ |  | $\alpha_{d_{2}}$ | $\alpha_{c}$ | $\alpha_{s}$ | $\alpha_{a}$ |
| $\beta_{0}$ | -1.12E+01 | 1.47E-01 | 2.15E-02 | 5.65E-03 | -1.86E-02 | -2.33E-02 | -5.74E-04 | -2.00E-06 | 1.09E-07 | 3.34E-03 | -9.66E-04 | -3.33E-03 |
| $\beta_{c}$ | -1.85E-01 | 1.36E-01 | 5.65E-03 | 1.84E-02 | -9.99E-04 | -2.44E-03 | -2.77E-05 | -5.07E-07 | 2.77E-08 | -3.18E-03 | -1.01E-03 | -8.52E-04 |
| $\beta_{s}$ | 1.78E+00 | 2.00E-01 | -1.86E-02 | -9.99E-04 | 4.00E-02 | 5.00E-03 | 8.28E-05 | 1.39E-06 | -7.60E-08 | -2.32E-03 | -4.33E-03 | 1.51E-03 |
| $\beta_{a}$ | 3.76E+00 | 4.73E-01 | -2.33E-02 | -2.44E-03 | 5.00E-03 | 2.24E-01 | -8.14E-03 | 2.11E-06 | -1.15E-07 | -4.98E-03 | 3.83E-03 | -1.41E-02 |
| $\beta_{e}$ | -5.79E-02 | 2.47E-02 | -5.74E-04 | -2.77E-05 | 8.28E-05 | -8.14E-03 | 6.08E-04 | 3.20E-08 | -1.75E-09 | -2.13E-04 | 1.70E-04 | 1.19E-03 |
|  | 2.66E-04 | 2.40E-05 | -2.00E-06 | -5.07E-07 | 1.39E-06 | 2.11E-06 | 3.20E-08 | 5.76E-10 | -3.14E-11 | -8.34E-07 | 1.79E-07 | 6.41E-07 |
| $\alpha_{d_{2}}$ | -1.45E-05 | 1.31E-06 | 1.09E-07 | 2.77E-08 | -7.60E-08 | -1.15E-07 | -1.75E-09 | -3.14E-11 | 1.72E-12 | 4.55E-08 | -9.76E-09 | -3.50E-08 |
| $\alpha_{c}$ | 3.08E-01 | 1.00E-01 | 3.34E-03 | -3.18E-03 | -2.32E-03 | -4.98E-03 | -2.13E-04 | -8.34E-07 | 4.55E-08 | 1.01E-02 | -5.02E-04 | -1.06E-03 |
| $\alpha_{s}$ | 4.37E-01 | 9.21E-02 | -9.66E-04 | -1.01E-03 | -4.33E-03 | 3.83E-03 | 1.70E-04 | 1.79E-07 | -9.76E-09 | -5.02E-04 | 8.49E-03 | 8.34E-04 |
| $\alpha_{a}$ | 1.05E+00 | 1.75E-01 | -3.33E-03 | -8.52E-04 | 1.51E-03 | -1.41E-02 | 1.19E-03 | 6.41E-07 | -3.50E-08 | -1.06E-03 | 8.34E-04 | 3.05E-02 |
| $\alpha_{e}$ | -1.06E-01 | 1.56E-02 | -4.42E-04 | -2.49E-04 | 4.56E-04 | 1.53E-03 | -9.57E-05 | 1.94E-07 | -1.06E-08 | -5.42E-05 | 4.42E-05 | -1.70E-03 |

1. Authors thank Drs. Hoffman and Apostoaei (ORRISK, Oak Ridge, TN, USA) for the parameterization of the onset function. [↑](#footnote-ref-1)
